# Editorial: Sensorimotor decoding: characterization and modeling for rehabilitation and assistive technologies, volume II

**DOI:** 10.3389/fnhum.2025.1619232

**Published:** 2025-06-03

**Authors:** Jean Faber, Vassiliy Tsytsarev, Miguel Pais-Vieira, Tetiana Aksenova

**Affiliations:** ^1^Neuroscience Division, Department of Neurology and Neurosurgery, Universidade Federal de São Paulo, Escola Paulista de Medicina, São Paulo, Brazil; ^2^School of Medicine, University of Maryland, Baltimore, MD, United States; ^3^Department of Medical Sciences iBiMED – Institute of Biomedicine, University of Aveiro, Aveiro, Portugal; ^4^Université Grenoble Alpes, CEA, LETI, Clinatec, Grenoble, France

**Keywords:** brain computer interface, assistive technology, rehabilitation, sensorimotor decoding, sensorimotor characterization

The dynamic field of Brain-Computer Interface (BCI) continues to promote crucial insights and technological advancements with significant implications for neurorehabilitation and assistive technologies. The present Research Topic, *Sensorimotor Decoding: Characterization and Modeling for Rehabilitation and Assistive Technologies Vol II* aims to increase our current understanding of sensorimotor processing in healthy individuals and in pathologies. Furthermore, it seeks to translate this knowledge to rehabilitation protocols, BCI procedures, and neurophysiological mechanisms through different approaches applied to the characterization, identification, and classification of electrophysiological sensorimotor patterns.

In the first volume (Pais-Vieira et al., 2023), the set of studies demonstrated that bridging the gap between basic and clinical sensorimotor science requires balancing time-proven techniques and novel technological approaches. Namely, encoding, decoding, and action were studied in normal and altered conditions using natural and artificial stimuli. In this second volume, the novel set of studies includes original articles as well as reviews and a case report, diversifying the types of approaches to study sensorimotor activity. These studies cover a range of topics from hardware design and algorithm development to clinical application. Collectively, they reflect an evolution in the field of BCI.

Specifically, Marzulli et al. investigated Phase-Amplitude Coupling (PAC) as features of Electrocorticography (ECoG) signals for classifying motor tasks in a tetraplegic patient. The authors demonstrated the ability of pseudo-online decoding of three different states (idle, left-hand, right-hand movements) with phase coupling, namely for theta/low-gamma and beta/high-gamma frequency bands.

Examining the basis of hardware characteristics, Saad et al. provided a comprehensive analysis of BCI decoding circuits, emphasizing the trade-offs between power consumption and performance. They reviewed the 2010–2025 literature on this issue and discussed quantitative metrics like Information Transfer Rate (ITR) and Classes per Second (CpS). They emphasized that energy efficiency should also receive attention for practical BCI implementation.

Furthermore, Radwan et al. addressed the overfitting problem in non-invasive Electroencephalography (EEG)-based BCIs for inner speech classification. The introduction of the 'BruteExtraTree' model offers a promising way for improving classification accuracy, considering the subject-dependence variability. Their study highlights the need for noise reduction and standardized EEG protocols.

The case report described by Ma et al. illustrates the technological advancements in the field of BCI in clinical applications. They demonstrated how Robot-Assisted Bimanual Task-Oriented Training (RABTOT) can help induce neuroplasticity related to functional upper limb movements in a patient with incomplete spinal cord injury. The observed correlation between the increase of Event-Related Desynchronization (ERD) in the sensorimotor cortex with the improvement in upper limb function provides evidence regarding the neurophysiological mechanisms underlying rehabilitation.

Exploring therapeutic interventions, Marques Dantas et al. in their narrative review highlighted the Laparoscopic Implantation of Neuroprosthesis (LION) as a novel neuromodulation technique for Spinal Cord Injury (SCI) rehabilitation. They show how electric stimulation performed on the lumbosacral nerve plexus can improve physiological functions and potentially promote neuroplasticity in patients with SCI. The authors also point toward the future integration of LION with BCI protocols to enhance rehabilitation outcomes.

Finally, the bibliometric analysis made by Liu et al., considering the period from 2004 to 2024, provided a comprehensive research BCI overview for rehabilitation. They outlined how the main Research Topics in this area have evolved, from EEG recording paradigms to other BCI applications in communication and motor control. This study works as an excellent guide for directing new research strategies in the BCI field.

**Figure 1:**
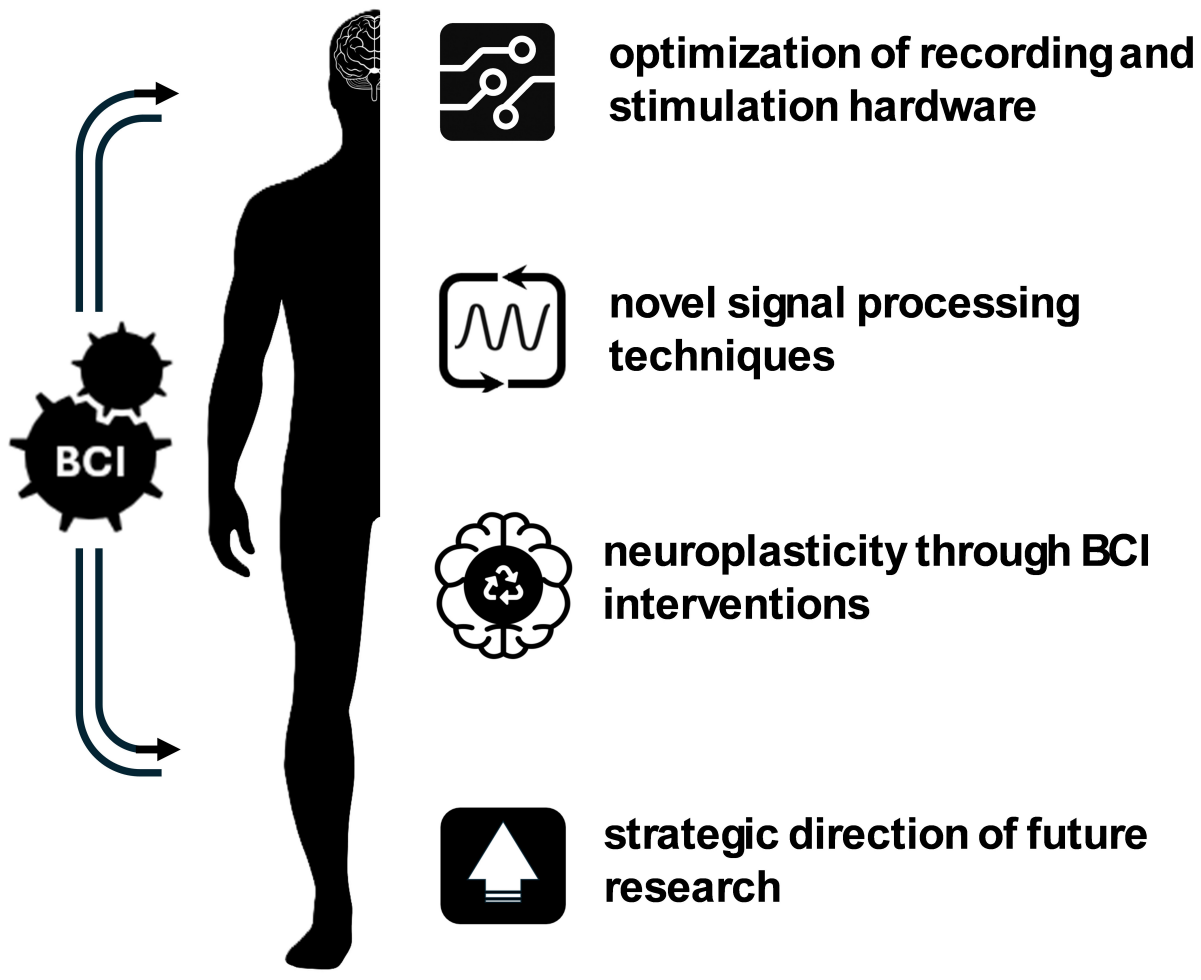
Directives for brain-computer interfaces on sensorimotor assistance and rehabilitation. Hardware optimization, signal processing innovation, BCI-guided neuroplasticity enhancement, and future research directions. This editorial shows the importance of these four aspects for developing the BCI area.

The results of the studies listed in this editorial emphasize the interdisciplinary nature of the BCI field for neurorehabilitation and assistive applications (Figure 1). It shows that the progress in BCI area relies on an integrated approach focusing on: (i) the optimization of recording and stimulation hardware—considering efficiency, biocompatibility, and sensor miniaturization; (ii) the development of novel signal processing techniques—considering more robust and adaptable algorithms for different contexts, as well as features that maximize neural code/decode information; (iii) the enhancement of neuroplasticity through BCI-guided therapeutic interventions—with personalized protocols featuring direct stimulation of neural pathways with hybrid technologies, and real-time feedback; and (iv) the strategic direction of future research—pointing toward new modalities of signal acquisition, bidirectional interfaces, and innovative applications. The integration of these guidelines is crucial for the development of more impactful and, at the same time, more accessible solutions in the BCI field. By consequence, the autonomy and quality of life of individuals with sensorimotor impairments will become increasingly effective.
